# Contraceptive use among women with kidney transplants in the United States

**DOI:** 10.1007/s40620-021-01181-0

**Published:** 2021-11-13

**Authors:** Silvi Shah, Annette L. Christianson, Shalini Bumb, Prasoon Verma

**Affiliations:** 1 Division of Nephrology, Kidney C.A.R.E. (Clinical Advancement, Research and Education) Program, University of Cincinnati, 231 Albert Sabin Way, MSB 6112, Cincinnati, OH 45267, USA; 2 Department of Environmental Health, University of Cincinnati, Cincinnati, OH, USA; 3 Cincinnati Children’s Hospital Medical Center, Cincinnati, OH, USA

**Keywords:** Rates, Contraception, Race/ethnicity, Kidney transplant

## Abstract

**Background:**

Kidney transplant improves reproductive function in women with end-stage kidney disease. Little is known about contraceptive use in women with history of kidney transplants.

**Methods:**

Using data from the United States Renal Data System, we evaluated for each calendar year women with kidney transplantation between 1/1/2005 and 12/31/2013 who were aged 15–44 years with Medicare as the primary payer and linked data from the United Network for Organ Sharing, for up to three entire years after the date of transplantation. We determined rates of contraceptive use and used multivariable logistic regression to identify factors associated with contraceptive use.

**Results:**

The study cohort included 13,150 women and represented 26,624 person-years. The rate of contraceptive use was 9.5%. Compared to women aged 15–24 years, contraceptive use was lower in women aged 30–34 years (OR 0.67; CI 0.58–0.78), 35–39 years (OR 0.36; CI 0.31–0.43), and 40–44 years (OR 0.23; CI 0.19–0.28). Compared to white women, contraceptive use was higher both in black women (OR 1.26; CI 1.10–1.43) and Native American women (OR 1.52; CI 1.02–2.26). Women had lower rates of contraceptive use in the second-year post-transplant (OR 0.87; CI 0.79–0.94) and the third-year post-transplant (OR0.69; CI 0.62–0.76) than in the first-year post-transplant. Women with a history of diabetes had a lower likelihood of contraceptive use (OR 0.80; CI 0.65–0.99).

**Conclusion:**

Among women with kidney transplants, contraceptive use remains low at 9.5%. Factors associated with a higher likelihood of contraceptive use include younger age and black and Native American race/ethnicity; and second- and third-year post-transplant. The history of diabetes is associated with a lower likelihood of contraceptive use. The study highlights the need of increasing awareness for safe and effective contraceptive use in women with kidney transplants.

## Introduction

Although end-stage kidney disease (ESKD) adversely impacts fertility, there is a return of reproductive function following a kidney transplant, and conception is common [1, 2]. History of kidney transplant increases the risk of adverse pregnancy outcomes, including pre-eclampsia, gestational hypertension, low birth weight babies, and preterm births [3, 4]. Women with kidney transplants are frequently on mycophenolate mofetil which is associated with increased risk of miscarriages and congenital anomalies of cleft lip, cleft palate, absent auditory canal, and microtia [1]. Therefore, provision of safe and effective contraception should be readily available especially to women with kidney transplants who take teratogenic medications like mycophenolate mofetil, and who wish to delay or avoid pregnancy.

Unplanned pregnancies are common in women with kidney transplants and have been reported to range from 30–50% in small cohort studies [5, 6]. Pre-pregnancy counseling discussing the effect of pregnancy on kidney disease and the impact of kidney disease on maternal and fetal outcomes in kidney transplant recipients is imperative. It is of paramount importance that pregnancies are planned, and effective methods of contraception are provided to women with history of kidney transplants to ensure that pregnancies do not occur prior to maternal optimization [7, 8]. Nephrologists do not usually discuss contraception with their female patients of childbearing age [9, 10]. Little is known about the contraceptive use and factors associated with its use among women with history of kidney transplants.

We used the national registry, United States Renal Data System (USRDS), to test our hypothesis that contraceptive use in women with kidney transplant remains low. The present study examines rates and factors associated with contraceptive use in women with history of kidney transplants, including race/ethnicity, age, type of immunosuppression, donor type, cause of ESKD, and socioeconomic status from a large cohort, which is not a voluntary registry.

## Methods

### Data sources and study population

**Fig. 1:**
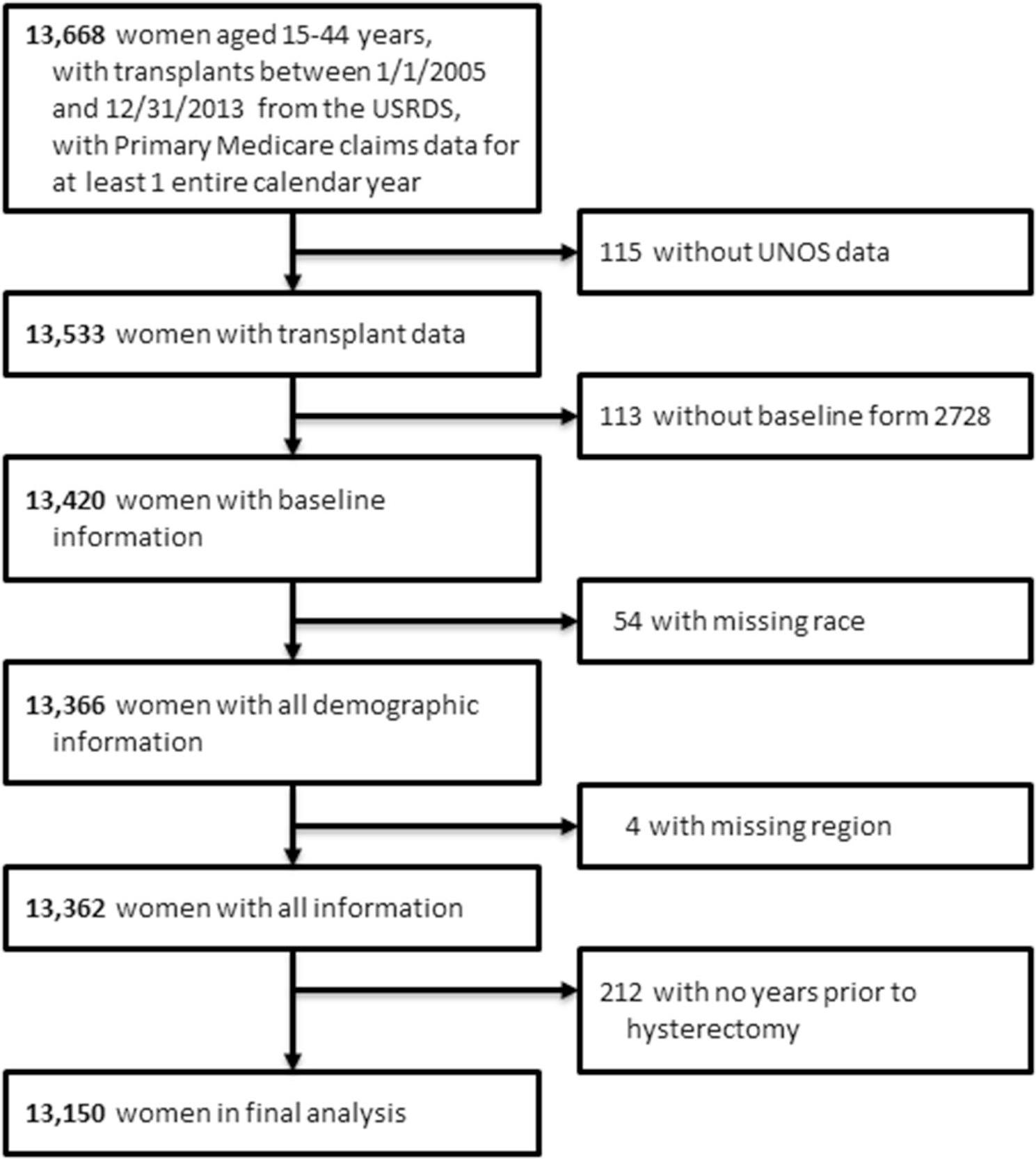
Cohort selection flow diagram

The USRDS database is a national registry of patients receiving chronic dialysis, and contains information from the ESKD Medical Evidence Form of the Centers for Medicare and Medicaid Services (CMS; form CMS-2728), as well as Medicare Part A institutional claims and Medicare Part B physician/supplier claims, and United Network for Organ Sharing (UNOS) registry data [11]. Using data from the United States Renal Data System covering 1/1/2005 through 12/31/2014, we included women who, for at least one entire year of the three following their transplant date between 2005 and 2013, fulfilled the following criteria: were aged 15–44 years, had history of kidney transplant, had primary Medicare claims data, and had linked data for UNOS in USRDS; if they had multiple years which qualified, the years were entered as separate observations for rates and models. The study included 26,624 person-years for 13,150 unique women. Information on the insurance coverage for the study period was obtained from the USRDS payer history file. Figure 1 illustrates the study cohort derivation. Due to use of the de-identified data, the University of Cincinnati Institutional Review Board committee deemed the study exempt from requiring informed consent.

We determined the contraceptive usage among women with kidney transplants in the United States. Using the ICD-9-CM diagnosis and procedure codes and current procedural terminology, 4th revision (CPT-4) codes, we searched for discharge diagnoses and medical procedures indicative of contraceptive use, which were then used to create binary indicators for each person-year for each type of contraception and for overall contraception among women with kidney transplants. Prior studies have validated the specificity of this code-based method to determine contraceptive use [12–14]. We categorized contraception into tubal ligation, intrauterine device, implant, diaphragm, injection, pill/others (oral contraceptives, spermicide, and female barrier methods), and emergency contraception according to the Medicare inpatient and outpatient claims (Supplement 1). We excluded women who were identified as having had a hysterectomy or bilateral oophorectomy in any study year for that year and all later years. We included women who were identified as having had a tubal ligation for that year but excluded them for all later years.

The patients’ files were used to obtain information on age, race/ethnicity, and the ESKD network. We categorized race/ ethnicity as Hispanic, non-Hispanic Asian, non-Hispanic black, non-Hispanic Native American, and non-Hispanic white. We categorized the ESKD networks into geographical regions of midwest, northeast, south, and west [15]. The CMS-2728 form was used to obtain information on the ESKD cause, body mass index, and comorbidities (diabetes, hypertension, and smoking history) [11]. We used the residence file combined with the zip code level data from the United States Census Bureau American Community Survey five-year estimates (2007 to 2011) to determine the neighborhood poverty level, defined by the percent of zip code residents living below the federal poverty level, and was grouped into five categories: I (< 13.8%), II (13.8–19.9%), III (20.0–39.9%), IV (40% or more), and unknown [16, 17]. The linked UNOS registry data were used to supply information on donor type, serum creatinine, and type of immunosuppression at transplant. Glomerular filtration rate (GFR) was calculated from serum creatinine values from six months after transplant from the UNOS follow-up file, using the Modification of Diet in Renal Disease equation, and was reported in ml/min/1.73 m^2^. For continuous measures of age, body mass index, and time on dialysis, groups were created based on clinical relevance. Patients with unavailable information on covariates were categorized into a “missing” group for that covariate.

### Statistical analysis

Percentages for categorical data and means ± standard deviations for continuous variables were used to present summary statistics. Differences between groups were tested using chi-squared tests for categorical variables, and *t* tests for continuous variables. Statistical significance was set at a two-tailed *p* value of 0.05, unadjusted for multiple tests. The unadjusted rates of contraceptive use were determined and expressed as the percent of person-years in which at least one indicator of contraceptive use appeared, for the overall cohort, and by race/ethnicity, age, calendar year of kidney transplant, and donor type. Multivariable logistic regression, with subject as a repeated effect, was used to determine the factors associated with contraceptive use. Multivariable logistic models were non-parsimonious, and included the covariates of age, race/ethnicity, body mass index, calendar year of kidney transplant, ESKD cause, neighborhood poverty level, geographical region, time on dialysis, year after transplant, comorbidities, history of smoking, GFR, donor type, and types of immunosuppression. Age, calendar year of kidney transplant, and neighborhood poverty level were determined from the beginning of each year post-transplant. Risk estimates for contraceptive use among kidney transplant recipients were expressed as odds ratios (ORs) and their 95% confidence intervals (95% CIs). The data were analyzed using SAS 9.4 (SAS Institute, Cary, NC).

## Results

### Baseline demographics and clinical characteristics

The study cohort included 26,624 observed person-years for 13,150 women.

Table 1 shows the characteristics of the women with history of kidney transplants separated by contraceptive use. The mean ages at study entry were 29 ± 7 years for women with any contraceptive use and 33 ± 8 years for women with no contraceptive use. Women with any contraceptive use had a higher proportion of younger women aged 15–34 years as compared to women without contraceptive use. With regard to race/ethnicity, women with any contraceptive use had a higher proportion of blacks (32.0% vs. 29.7%) and lower proportion of whites (40.3% vs. 43.4%) than did women without contraceptive use. Women without contraceptive use had higher rates of diabetics as compared to those without contraceptive use (18.0% vs. 15.3%).

### Contraceptive rates in women with kidney transplants

The rate of any contraceptive use during the first three years post-transplant was 9.5%.

**Fig. 2:**
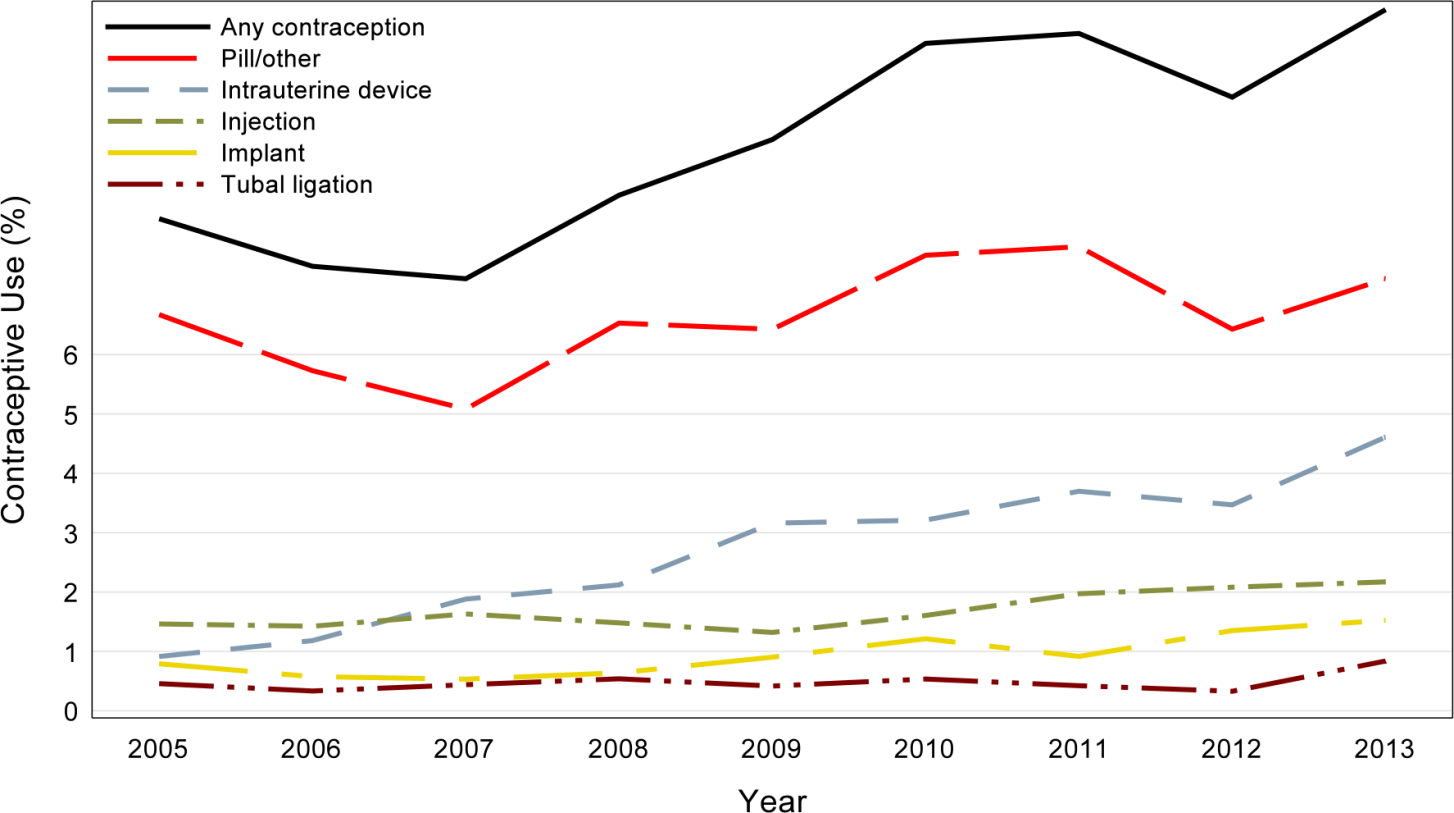
Rates of types of contraceptive use among women with kidney transplant from 2005–2013. Any contraception shows a fairly steady increase across the study time; all categories increase or remain constant from beginning to end. *Emergency contraception and diaphragm had rates of less than 0.1% for all years, not shown in the graph

Overall, rates of different types of contraceptive use were as follows: intrauterine device insertions (2.6%), injection (1.7%), implant (0.9%), tubal ligation (0.5%), diaphragm (0.1%), emergency contraception (< 0.1%), and pills/others (6.6%). Contraceptive use increased from 2005 to 2013 (8.3% vs. 11.8%). From 2005 to 2013, there was an increase in the rate of intrauterine device insertions from 0.9% to 4.6%, implant insertion from 0.8% to 1.5%, tubal ligation from 0.5% to 0.8%, injection from 1.5% to 2.2%, and pills/others from 6.7% to 7.3% (Fig. 2).

**Fig. 3:**
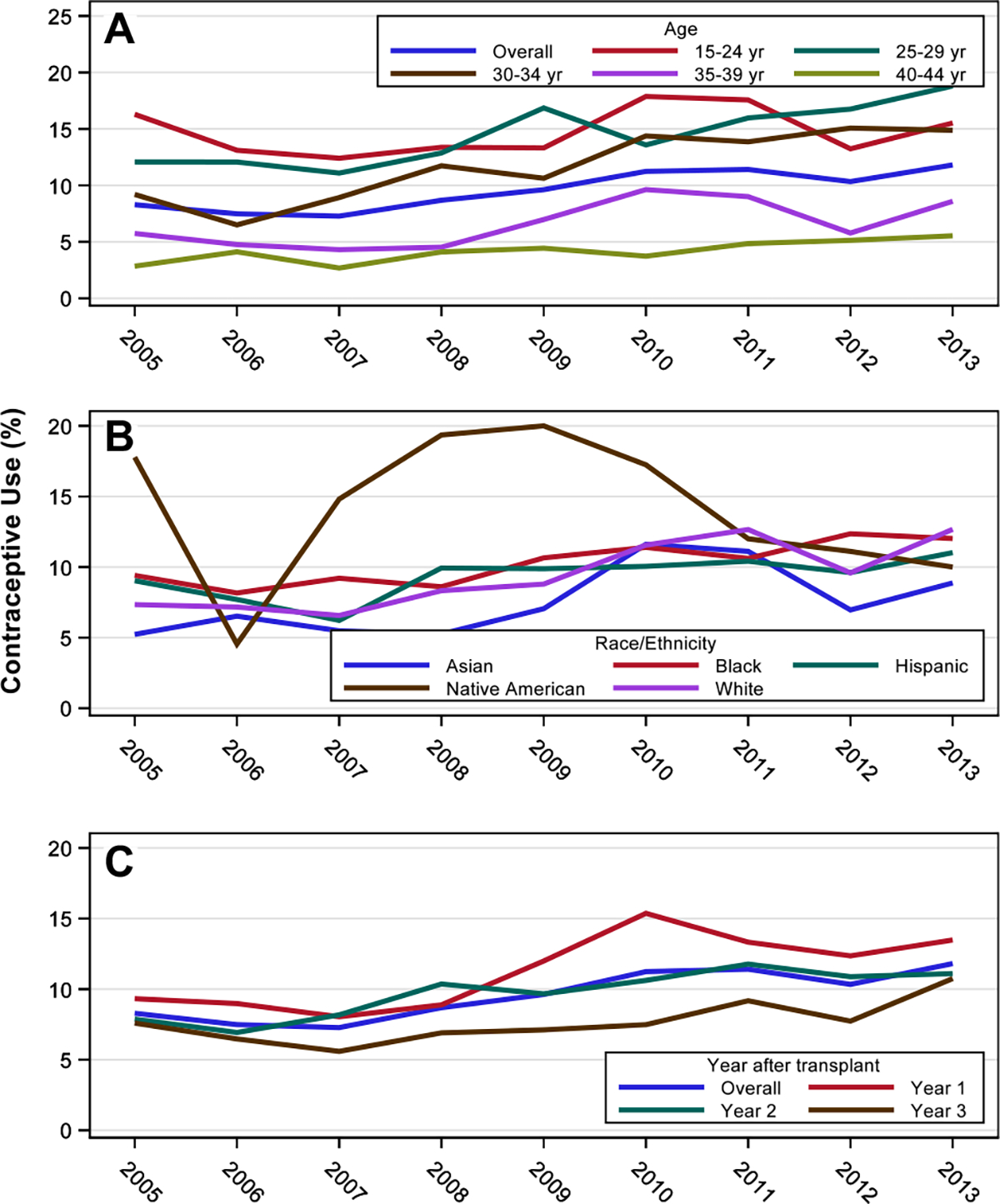
Contraceptive rates in women with kidney transplant by (**A**) age, **B** race, **C** post-transplant year. All three graphs show an upward trend from 2005 to 2013. The age graph shows a decline in rates with increasing age. The race graph shows a higher rate among Native Americans as compared to other races. The post-transplant year graph shows the highest rate of contraceptive use in the first post-transplant year

Among women with kidney transplants, with regard to race/ethnicity, Native Americans had the highest rate of contraceptive use (14.0%) followed by blacks (10.2%), Hispanics (9.3%), whites (9.1%), and Asians (7.5%). There was an increase in contraceptive use from 2005–2013 among all races except Native Americans: among Asians from 5.2% to 8.9%, among blacks from 9.4% to 12.0%, among Hispanics from 9.0% to 11.0%, and among whites from 7.3% to 12.7% (Fig. 3a) There was an increase in the rate of contraceptive use from 2005 to 2013 across all age groups except among women aged 15–24 years. Contraceptive use was highest among women aged 15–24 years (14.6%), followed by women aged 25–29 years (14.2%), and lowest among women aged 40–44 years (4.1%) (Fig. 3b). Figure 3c shows the rates of contraceptive use by post-transplant year. Among women with kidney transplant, contraceptive use increased from 9.3% in 2005 to 13.5% in 2013 for the first-year post-transplant, from 7.9% to 11.1% for the second-year post-transplant, and from 7.6% to 10.7% for the third-year post-transplant.

### Factors associated with contraceptive use in women with kidney transplant

**Fig. 4:**
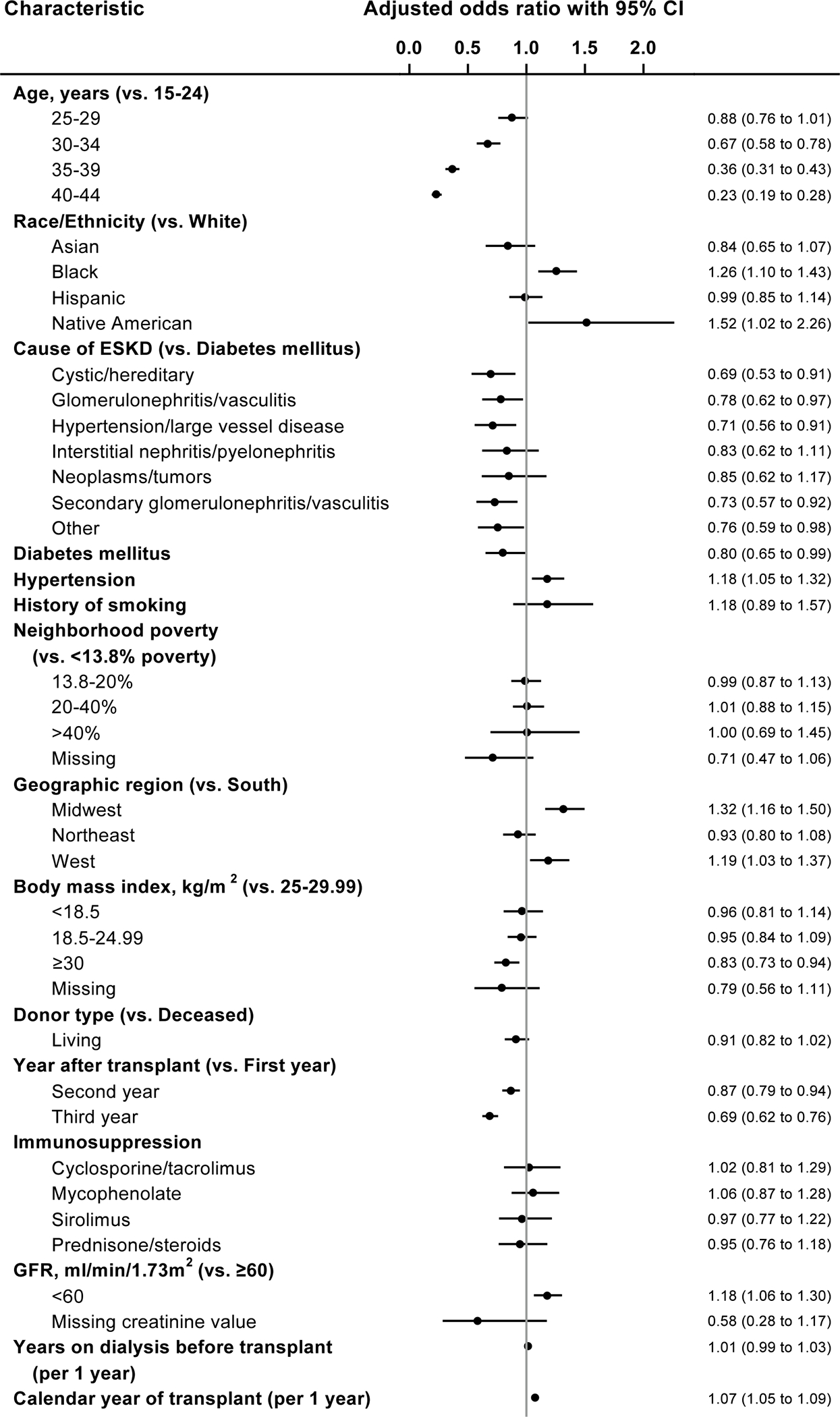
Factors associated with contraceptive use in women with kidney transplant. *ESKD end stage kidney disease

Figure 4 elucidates the factors associated with likelihood of contraceptive use among women with ESKD from the adjusted logistic regression model. Compared to women aged 15–24 years, contraceptive use was lower in women aged 30–34 years (OR 0.67; CI 0.58–0.78), 35–39 years (OR 0.36; CI 0.31–0.43), and 40–44 years (OR 0.23; CI 0.19–0.28). As compared to white women, contraceptive use was higher in black women (OR 1.26; CI 1.10–1.43) and Native American women (OR 1.52; CI 1.02–2.26). There was an upward linear trend associated with calendar year of kidney transplant (OR 1.07 for one-year increase; CI 1.05–1.09). Among kidney transplant recipients, as compared to women with ESKD due to diabetes, there was a lower likelihood of contraceptive use in women with ESKD due to cystic disease (OR, 0.69; CI, 0.53–0.91), ESKD due to glomerulonephritis (OR 0.78; CI 0.62–0.97), ESKD due to hypertension/large vessel disease (OR 0.71; CI 0.56–0.91), and ESKD due to secondary glomerulonephritis/ vasculitis (OR 0.73; CI0.57–0.92). As compared to women residing in the southern geographical region, contraceptive use was higher in women residing in the midwestern region (OR 1.32; CI 1.16–1.50) and western region (OR, 1.19; CI, 1.03–1.37). Women receiving kidney transplant had a lower likelihood of contraceptive use in the second post-transplant year (OR 0.87; CI 0.79–0.94) and the third post-transplant year (OR 0.69; CI 0.62–0.76) than in the first post-transplant year. Compared to women with body mass index 25.1–30 kg/m^2^, contraceptive use was lower in women with body mass index > 30 kg/m^2^ (OR 0.83; CI 0.73–0.94). The comorbidity of diabetes was associated with a lower likelihood of contraceptive use (OR 0.80; CI 0.65–0.99). Compared to women with GFR of 60 ml/min/1.73 m^2^ and greater, contraceptive use was higher among women with GFR < 60 ml/min/1.73 m^2^ (OR 1.18; CI 1.06–1.30). History of smoking, immunosuppression type, donor type, neighborhood poverty level, hypertension, and years on dialysis pre-transplant were not significantly associated with contraceptive use among women with kidney transplants.

## Discussion

Our study is the largest study to determine the rates and factors associated with contraceptive use in women with history of kidney transplants. Using a national registry of patients with ESKD, we found that although contraceptive use rates increased in the last decade, overall contraceptive use (other than male condoms) was low at 9.5% among women during the first three years after receiving a kidney transplant. Age, race/ethnicity, ESKD cause, year of conception post-transplant, history of diabetes, and GFR were important factors associated with contraceptive use in the current study.

The present study reports a low rate of contraceptive use in women with history of kidney transplants in the United States. Currently, no published contemporary data exist for contraceptive use among kidney transplant recipients. A recent study showed that among women with end-stage kidney disease undergoing dialysis, only 5.3% used contraception [18]. In contrast, 65% of women in the general population in the United States used a method of contraception between 2015 and 2017 that increased with age [19]. The low rate of contraceptive use among women with kidney transplants may be attributed to lack of awareness about return of fertility and inadequate counseling regarding contraception to prevent unplanned pregnancies. About one-third of pregnancies in kidney transplant recipients are unplanned [20]. A survey by French et al. reported that less than half of the kidney transplant recipients received counseling about contraception [21]. The reasons for not providing reproductive counseling include lack of training, inadequate confidence, and a perceived lack of evidence in the field [22]. Also, due to the low rate of pregnancy in the kidney transplant population of ~ 14 per 1000 person-years, it is possible that patients, rather than not using effective contraception, are directed towards the use of barrier methods including condoms (not captured in the present study), which are not the safest contraceptive device, but the one that is likely recommended by the gynecologist due to inadequate experience on the use of contraceptives in kidney transplant recipients.^2^

Our study shows the higher likelihood of contraceptive use in black women and Native American women as compared to white women. In contrast, in the general population, there is a higher percentage of contraceptive use in non-Hispanic white women as compared to non-Hispanic black women (67% vs. 60%) [19]. These racial/ethnic differences in contraceptive use in the kidney transplant population may be due to different cultural beliefs, social values, family support systems, and health literacy. Also, high-income women may have received prescription through channels not identified by the records in the present study [23]. Although we took into account socioeconomic status, we were not able to determine health literacy, which remains a limitation of the present study.

Among women with kidney transplants, ESKD caused by cystic disease, or glomerulonephritis was associated with lower odds of contraceptive use as compared to diabetes. In transplant recipients, pregnancy is more likely in women with ESKD due to cystic disease or to glomerulonephritis compared to women with ESKD due to diabetes [2].

While reasons remain unclear, we speculate that this may be related to the fact that women with ESKD due to glomerulonephritis or cystic disease are overall healthier with return of fertility following a kidney transplant than those having ESKD due to diabetes.

Women in the second and third post-transplant year had a lower likelihood of contraceptive use than did women in the first post-transplant year. According to the American Society of Transplantation guidelines, the ideal time of conception is between 1 and 2 years after kidney transplantation, whereas the European best practice guidelines recommend delaying conception for a period of 2 years after transplantation [24, 25]. Therefore, the need to avoid pregnancies in the first-year post-transplant may have led to a higher rate of contraceptive use in the first-year post-transplant year than in the second and the third post-transplant year.

A significant strength of our study is that it includes women of childbearing age with kidney transplant in the United States, thus providing us with accurate information on contraceptive use in a heterogeneous population of kidney transplant recipients. Since USRDS captures all patients with Medicare claims, the present study gives us accurate estimates of contraceptive use other than the male condoms in the first three years post-transplant among women. Additionally, we have identified factors that are associated with likelihood of contraceptive use. Our study has some limitations. First, due to the observational design of our study, we are unable to identify interventions that may possibly affect contraceptive practices among women with kidney transplants. Second, we are unable to determine exact rates for oral contraceptive use (others/pills category in the present study) due to lack of pharmacy claims data. Third, we are not able to assess the impact of contraceptive methods (such as intrauterine device or injection) that can last for subsequent years. Fourth, we are not able to identify contraceptive methods that may have been used by men including the use of condoms. Fifth, we were unable to compare contraceptive use in an age-matched non-transplant population due to use of observational de-identified data.

In conclusion, our study demonstrates lower rates of contraceptive use in women with kidney transplant in the United States, significant differences across race/ethnicity in contraceptive use, and the association of post-transplant year on the likelihood of contraceptive use among kidney transplant recipients. The present study underscores the importance for nephrologists to include contraception in reproductive counseling for women with kidney transplants that may lead to higher contraceptive use and possibly prevent unintentional pregnancies.

## Supplementary Material

Supplementary Material

## Figures and Tables

**Table 1:** Baseline characteristics of women with kidney transplant separated by contraceptive use during the follow-up period

Characteristics	Without recorded data on contraception *N* = 11,193	With recorded data on contraception *N* = 1957	*p* value
Demographics			
Age (years)^a^^b^	33 (8)	29 (7)	< 0.001
15–24	15.4	25.9	< 0.001
25–29	13.6	24.0	
30–34	19.7	24.4	
35–39	25.9	16.9	
40–44	25.4	8.8	
Race/ethnicity			0.001
Asian	5.7	4.5	
Black	29.7	32.0	
Hispanic	20.3	21.7	
Native American	1.0	1.6	
White	43.4	40.3	
Body mass index (kg/m^2^)^a^	26.5 (7.4)	25.7 (6.8)	< 0.001
< 18.5	8.8	10.5	< 0.001
18.5–25	39.5	42.0	
25.1–30	22.2	21.9	
> 30	25.6	23.1	
Missing	3.9	2.5	
Cause of ESKD			0.005
Cystic/hereditary	8.6	8.5	
Diabetes mellitus	19.0	16.5	
Glomerulonephritis	28.0	29.8	
Hypertension/large vessel disease	12.8	10.7	
Interstitial nephritis/pyelonephritis	5.0	6.0	
Malignancy	2.8	3.4	
Secondary glomerulonephritis/vasculitis	15.1	15.5	
Others	8.7	9.6	
Time on dialysis pre-transplant (years)	3.0 (3.1)	3.0 (2.9)	0.679
Neighborhood poverty level^b^ 0.119			
< 13.8%	59.8	58.3	
13.8–20%	18.1	18.6	
> 20–40%	18.8	20.2	
> 40%	1.4	1.7	
Missing	1.8	1.2	
Geographical region < 0.001			
Midwestern	22.8	26.5	
Northeastern	18.3	15.8	
Southern	39.4	36.9	
Western	19.6	20.8	
Comorbidities			
Diabetes mellitus	18.0	15.3	0.004
Hypertension/large vessel disease	72.1	72.7	0.585
Smoking	3.0	3.1	0.862
Donor type 0.069			
Living donor	34.9	32.8	
Deceased donor	65.1	67.2	
Year after transplant < 0.001			
Year 1	65.2	79.3	
Year 2	16.9	13.6	
Year 3	17.9	7.1	
Immunosuppression at the time of transplant			
Cyclosporine/tacrolimus	94.7	94.9	0.780
Mycophenolate	90.0	90.3	0.730
Sirolimus	7.0	6.2	0.209
Steroids/prednisone	94.0	94.0	0.989
GFR at 6 months after transplantation (ml/min/1.73 m^2^)^a^	62.7 (25.0)	63.6 (22.5)	0.118
≥ 60	48.6	47.4	0.094
< 60	50.5	52.1	
Missing	0.9	0.5	

a Reported in mean (standard deviation); all others are reported as percentages of person-years. *ESKD* end stage kidney disease

b Values associated with women’s first eligible year for study
